# The prognostic value of lncRNA SNHG4 and its potential mechanism in liver cancer

**DOI:** 10.1042/BSR20190729

**Published:** 2020-01-31

**Authors:** Yan Jiao, Yanqing Li, Baoxing Jia, Qingmin Chen, Guoqiang Pan, Fang Hua, Yahui Liu

**Affiliations:** 1 Department of Hepatobiliary and Pancreatic Surgery, The First Hospital of Jilin University, Changchun, Jilin 130021, P.R. China; 2 Department of Pathophysiology, College of Basic Medical Sciences, Jilin University, Changchun, Jilin 130021, P.R. China; 3 Department of Gastrointestinal Surgery, The First Hospital of Jilin University, Changchun, Jilin, 130021, P.R. China; 4 Cardiovascular Internal Medicine, The First Hospital of Jilin University, Changchun, Jilin 130021, P.R. China

**Keywords:** Liver cancer, prognosis, Small Nucleolar RNA Host Gene 4, SNHG4, The Cancer Genome Atlas

## Abstract

Background and object: Emerging evidence shows that non-coding RNA functions as new gene regulators and prognostic markers in several cancers, including liver cancer. Here, we focused on the small nucleolar RNA host gene 4 (SNHG4) in liver cancer prognosis based on The Cancer Genome Atlas (TCGA) data.

Methods: The expression data and clinical information were downloaded from TCGA. Chi-square tests evaluated the correlation between SNHG4 expression and clinical parameters. Differences in survival between high and low expression groups (optic cutoff value determined by ROC) from Cox regression analysis were compared, and *P*-value was calculated by a log-rank test. Kaplan–Meier curves were compared with the log-rank test. GSEA and ceRNA network were conducted to explore the potential mechanism.

Results: Data mining of lncRNA expression data for 371 patients with primary tumor revealed overexpression of SNHG4 in liver cancer. High SNHG4 expression was correlated with histological type (*P* = 0.01), histologic grade (*P* = 0.001), stage (*P* = 0.01), T classification (*P* = 0.004) and survival status (*P* = 0.013). Patients with high SNHG4 expression had poor overall survival and relapse-free survival compared with those with low SNHG4 expression. Multivariate analysis identified SNHG4 as an independent prognostic factor of poor survival in liver cancer. GSEA revealed related signaling pathway and ceRNA network explored the further mechanism.

Conclusion: High SNHG4 expression is an independent predictor of poor prognosis in liver cancer.

## Introduction

Liver cancer is one of the most digestive system malignancies in the world [1]. The 5-year survival rate has not improved in spite of recent advance in the treatment of liver cancer. The clinicians apply the histological classification and some biomarkers, such as Ki67, CD34, and AFP, to evaluate patients’ prognosis in the nowadays. However, there is also a challenge for clinicians make a judgment of liver cancer patients’ prognosis. Novel molecular classification for prognosis is urgent.

Non-coding RNA has attracted much attention recently. There have been too many researches about long non-coding RNA and microRNA in the field of oncology. Also, some molecular has been recognized as novel biomarkers for prognosis, including XIST [2], PVT1 [3], and MALT1 [4]. Small nucleolar RNA host gene 4 (SNHG4), a novel non-coding RNA, has first reported in the research of directly irradiated and bystander cells and was found to be up-regulated in irradiated TK6 cells but were repressed in bystander cells [5]. A recent study has revealed that lncRNA SNHG4 was associated with poor survival and recurrence in human osteosarcoma [6].

However, the prognostic role and potential mechanism of SNHG4 in liver cancer remain unclear. In the present study, we explored the SNHG4 expression in liver cancer and compared the relationship between SNHG4 expression and clinical parameters. In the further, we analyzed the effect of SNHG4 on the overall survival and relapse-free survival and made subgroup analysis to explore it in depth. Besides, we studied the related signaling pathway through GSEA and conducted the SNHG4-related ceRNA network.

## Materials and methods

### Data mining of TCGA database

SNGH4 expression pattern and its prognostic significance were validated from liver cancer tissues paired with normal liver tissues from the TCGA database by RNAseq (IlluminaHiSeq). The optic value of SNGH4 determined by ROC was used as a cutoff for defining the high and low SNGH4 expression groups. HCCDB database (http://lifeome.net/database/hccdb/home.html) was used to validate the results.

### Statistical analysis

Statistical analysis was performed using R (version 3.5.1) [7] and R packages including ggplot2 [8], pROC [9], and survival [10]. Patients were divided into two groups (SNHG4 high expression and SNHG4 low expression) by the proper threshold in ROC. Chi-squared tests were applied to assess the association between SNGH4 expression and clinicopathological features. Kaplan–Meier curve compared the overall/relapse-free survival between high and low SNHG4 expression groups with the log-rank test. The independent prognostic value of SNHG4 expression on liver cancer was assessed by univariate and subsequent multivariate Cox regression analysis. Differences were considered significant when *P* < 0.05.

### GSEA

GSEA is a computational method that determines whether an *a priori* defined set of genes shows statistically significant, concordant differences between two biological states [11,12]. In the present study, GSEA was performed by using the GSEA software 3.0 from the Broad Institute. The gene expression data were RNAseq data from TCGA-LIHC. The gene set of “h.all.v6.2.symbols.gmt”, which summarizes and represents specific, well-defined biological states or processes, was downloaded from the Molecular Signatures Database (http://software.broadinstitute.org/gsea/ msigdb/index.jsp). The normalized enrichment score (NES) was acquired by analyzing with permutations for 1000 times. Gene sets with normal *P*-value < 0.05 and false discovery rate (FDR) < 0.25 were considered to be significantly enriched.

### Conduction of ceRNA network

Differentially expression microRNAs and encoding genes between high SNHG4 group and low SNHG4 group were analyzed using limma packages [13], and adjust *P* value < 0.05 is presented as significant. The predicted miRNA–mRNA interactions were obtained from starbase v2.0 (http://starbase.sysu.edu.cn/starbase2/index.php) by defeat options [14,15]. The ceRNA network was conducted by merging DEMs, DEGs, and miRNA–mRNA interactions.

### Enrichment analysis

Gene Ontology (GO) function and Kyoto Encyclopedia of Genes and Genomes (KEGG) pathway enrichment analysis were performed using the R language Cluster profiler package [16]. The cluster Profiler is a Bioconductor software package that provides statistical analysis of functional clustering of gene sets.

## Results

### Patient characteristics

The demographic and clinicopathological characteristics gene expression data of 371 patients with liver cancer are analyzed and shown in Table 1.

**Table 1:** Baseline charateristics of patients with liver cancer

Characteristics	Number of patients (%)
Age	
<55	117(31.62)
≥55	253(68.38)
Gender	
FEMALE	121(32.61)
MALE	250(67.39)
Histological type	
Fibrolamellar Carcinoma	3(0.81)
Hepatocellular Carcinoma	361(97.3)
Hepatocholangiocarcinoma	7(1.89)
Histologic grade	
G1	55(14.82)
G2	177(47.71)
G3	122(32.88)
G4	12(3.23)
NA	5(1.35)
Stage	
I	171(46.09)
II	86(23.18)
III	85(22.91)
IV	5(1.35)
NA	24(6.47)
T classification	
T1	181(48.79)
T2	94(25.34)
T3	80(21.56)
T4	13(3.5)
TX	1(0.27)
NA	2(0.54)
N classification	
N0	252(67.92)
N1	4(1.08)
NX	114(30.73)
NA	1(0.27)
M classification	
M0	266(71.7)
M1	4(1.08)
MX	101(27.22)
Radiation therapy	
NO	338(91.11)
YES	8(2.16)
NA	25(6.74)
Residual tumor	
R0	324(87.33)
R1	17(4.58)
R2	1(0.27)
RX	22(5.93)
NA	7(1.89)
Vital status	
DECEASED	130(35.04)
LIVING	241(64.96)
Relapse	
NO	179(56.29)
YES	139(43.71)
SNHG4	
High	75(20.22)
Low	296(79.78)

### SNHG4 expression and association with clinicopathological variables

**Figure 1:**
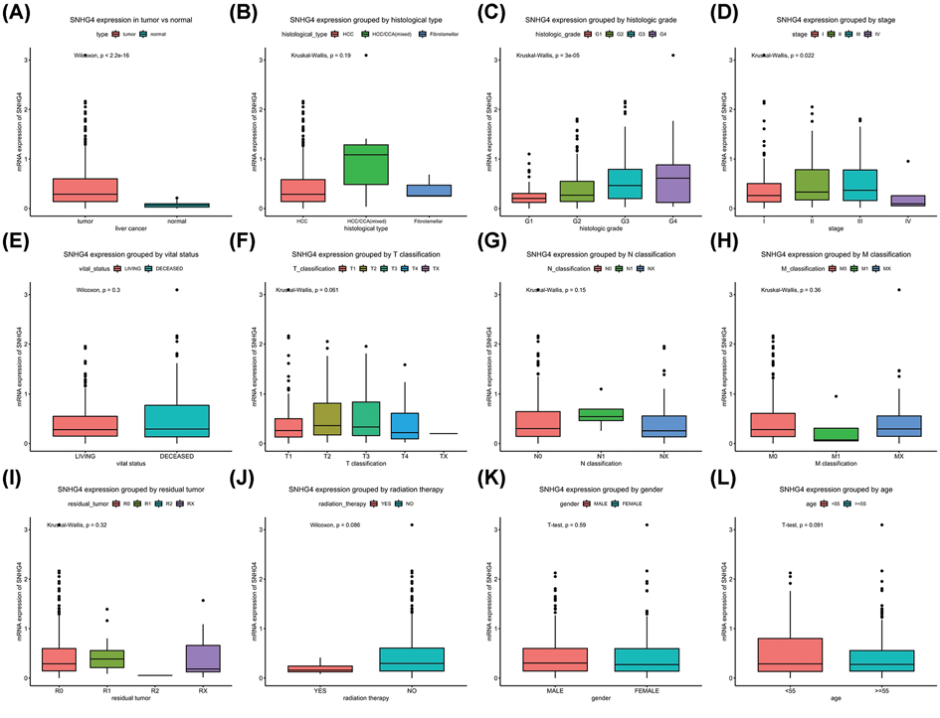
SNHG4 expression in liver cancer SNHG4 expression in liver cancer versus normal tissue (**A**) and grouped by histological type (**B**), histologic grade (**C**), stage (**D**), vital status (**E**), TNM classification (**F,G,H**), residual tumor (**I**), radiation therapy (**J**), gender (**K**), and age (**L**).

SNHG4 expression was significantly higher in liver cancer tissues (*n* = 371; *P* < 0.05) compared with normal liver tissues (*n* = 50), which was validated by HCCDB database (Supplementary Figure S1). Furthermore, significant differences in SNHG4 expression were found based on histological grade and stage (Figure 1). The patients with liver cancer were divided into high and low SNHG4 expression groups. And their clinicopathological parameters and survival outcomes were described in Table 2. The results proved a correlation between high SNHG4 expression and histological type (*P* = 0.01), histologic grade (*P* = 0.001), stage (*P* = 0.01), T classification (*P* = 0.004), and survival status (*P* = 0.013).

**Table 2:** The correlation between SNHG4 expression and clinical parameters

Clinical characteristics	Variable	No. of patients	SNHG4 expression				χ2	*P*-value
			High	%	Low	%		
Age	<55	117	31	(41.33)	86	(29.15)	3.5593	0.059
	≥55	253	44	(58.67)	209	(70.85)		
Gender	FEMALE	121	28	(37.33)	93	(31.42)	0.7023	0.402
	MALE	250	47	(62.67)	203	(68.58)		
Histological type	Fibrolamellar	3	0	(0)	3	(1.01)	12.2954	**0.010**
	Hepatocellular	361	70	(93.33)	291	(98.31)		
	Hepatocholangiocarcinoma	7	5	(6.67)	2	(0.68)		
Histologic grade	G1	55	4	(5.41)	51	(17.47)	16.3367	**0.001**
	G2	177	29	(39.19)	148	(50.68)		
	G3	122	37	(50)	85	(29.11)		
	G4	12	4	(5.41)	8	(2.74)		
Stage	I	171	23	(32.39)	148	(53.62)	10.4078	**0.010**
	II	86	23	(32.39)	63	(22.83)		
	III	85	24	(33.8)	61	(22.1)		
	IV	5	1	(1.41)	4	(1.45)		
T classification	T1	181	23	(30.67)	158	(53.74)	13.4311	**0.004**
	T2	94	26	(34.67)	68	(23.13)		
	T3	80	23	(30.67)	57	(19.39)		
	T4	13	3	(4)	10	(3.4)		
	TX	1	0	(0)	1	(0.34)		
N classification	N0	252	54	(72)	198	(67.12)	0.7889	0.546
	N1	4	1	(1.33)	3	(1.02)		
	NX	114	20	(26.67)	94	(31.86)		
M classification	M0	266	57	(76)	209	(70.61)	1.0165	0.497
	M1	4	1	(1.33)	3	(1.01)		
	MX	101	17	(22.67)	84	(28.38)		
Radiation therapy	NO	338	69	(100)	269	(97.11)	0.9617	0.327
	YES	8	0	(0)	8	(2.89)		
Residual tumor	R0	324	66	(89.19)	258	(88.97)	0.4091	0.959
	R1	17	3	(4.05)	14	(4.83)		
	R2	1	0	(0)	1	(0.34)		
	RX	22	5	(6.76)	17	(5.86)		
Survival status	DECEASED	130	36	(48)	94	(31.76)	6.2408	**0.013**
	LIVING	241	39	(52)	202	(68.24)		

Bold term represents *P*<0.05.

### High SNHG4 expression as an independent prognostic factor for poor overall survival

**Figure 2:**
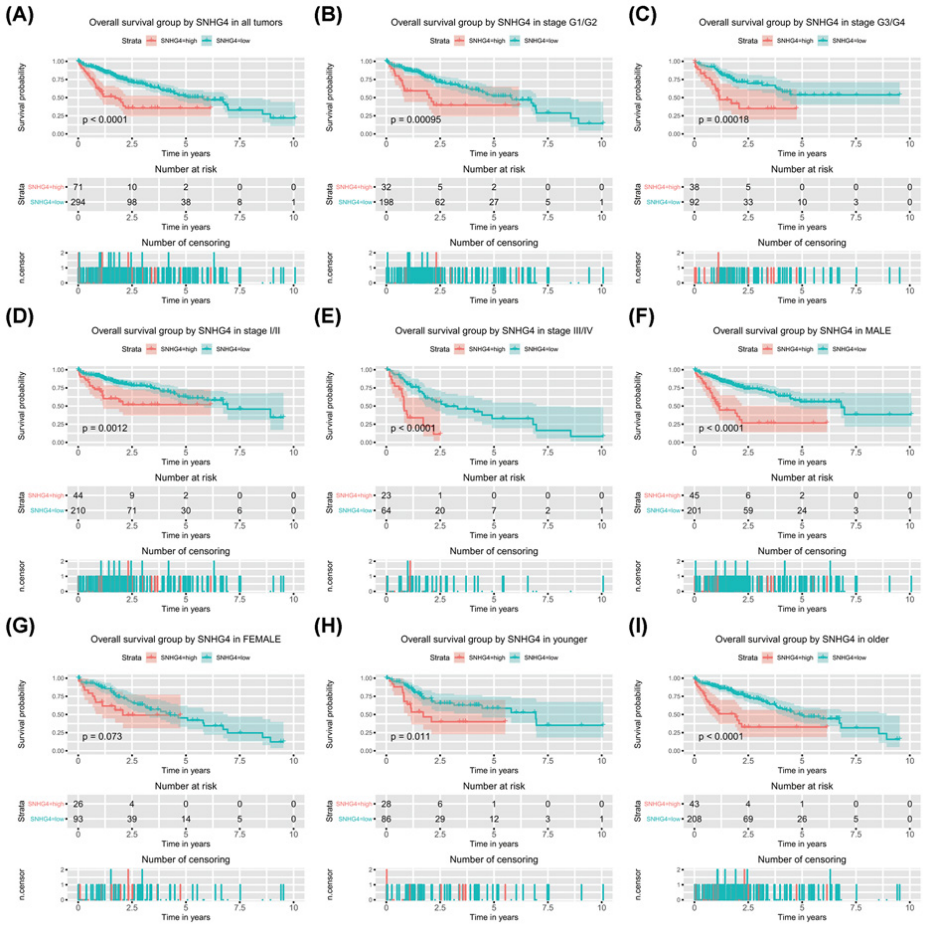
High SNHG4 expression is correlated with shorter overall survival (OS) High SNHG4 expression is correlated with shorter OS in patients with liver cancer (**A**) and the group of G1/G2 (**B**), G3/G4 (**C**), stage I/II (**D**), stage III/IV (**E**), male (**F**), female (**G**), younger (**H**), and older (**I**).

High SNHG4 expression was associated with poor overall survival (*P* < 0.0001; Figure 2), which was validated by HCCDB database (Supplementary Figure S2). Subsequent subgroup analysis proved that high SNHG4 expression was associated with poor overall survival of patients in all subgroup except for female (*P* = 0.073; Figure 2). According to univariate analysis, stage, T classification, residual tumor, and SNHG4 expression were associated with poor overall survival (Table 3). Further multivariate analysis determined the independent prognostic value of high SNHG4 expression for poor overall survival of liver cancer (hazard ratio: 2.84, 95% confidence interval: 1.90–4.23, *P* < 0.001; Table 3).

**Table 3:** Univariate and multivariate cox analysis of overall survival

Parameters	Univariate analysis			Multivariate analysis		
	Hazard ratio	95%CI (lower∼upper)	*P-*value	Hazard ratio	95%CI (lower-upper)	*P-*value
Age	1.02	0.7–1.48	0.926			
Gender	0.82	0.57–1.16	0.263			
Histological type	0.98	0.27–3.63	0.982			
Histologic grade	1.05	0.85–1.31	0.651			
Stage	1.38	1.15–1.65	0.001	0.86	0.68–1.07	0.175
T classification	1.65	1.38–1.98	0.000	1.84	1.45–2.34	0.000
N classification	0.71	0.5–1.03	0.071			
M classification	0.70	0.48–1.02	0.061			
Radiation therapy	0.52	0.26–1.03	0.061			
Residual tumor	1.42	1.12–1.79	0.004	1.44	1.13–1.85	0.003
SNHG4	2.83	1.91–4.2	0.000	2.83	1.9–4.23	0.000

### High SNHG4 expression as an independent prognostic factor for poor relapse-free survival

**Figure 3:**
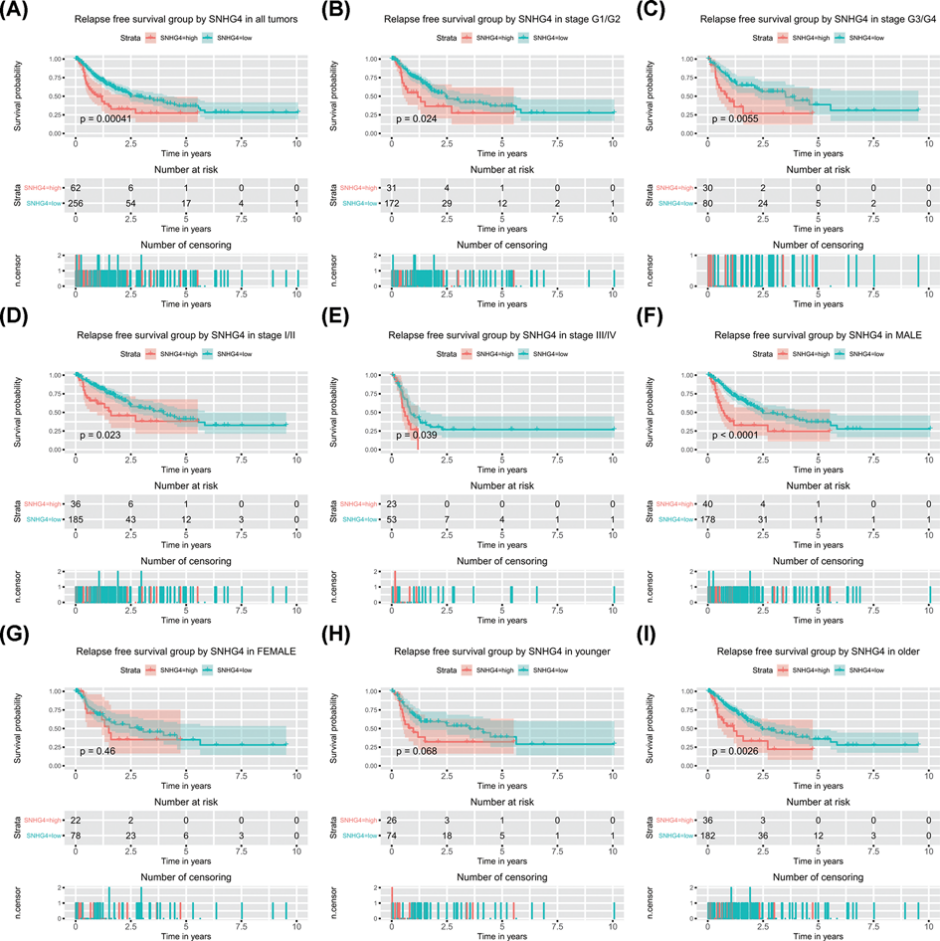
High SNHG4 expression is correlated with shorter relapse-free survival (RFS) High SNHG4 expression is correlated with shorter RFS in patients with liver cancer(**A**) and the group of G1/G2 (**B**), G3/G4 (**C**), stage I/II (**D**), stage III/IV (**E**), male (**F**), female (**G**), younger (**H**), and older (**I**).

High SNHG4 expression was associated with poor relapse-free survival (*P* < 0.001; Figure 3). Subsequent subgroup analysis proved that high SNHG4 expression was associated with poor relapse-free survival of patients in all subgroup except for female (*P* = 0.46; Figure 3) and younger (*P* = 0.068; Figure 3). According to univariate analysis, stage, T classification, residual tumor, and SNHG4 expression were associated with poor relapse-free survival (Table 4). Further multivariate analysis determined the independent prognostic value of high SNHG4 expression for poor relapse-free survival of liver cancer (hazard ratio: 1.95, 95% confidence interval: 1.29–2.94, *P* = 0.002; Table 4).

**Table 4:** Univariable and multivariable cox analysis of relapse-free survival

Parameters	Univariate analysis			Multivariate analysis		
	Hazard ratio	95%CI (lower∼upper)	*P-*value	Hazard ratio	95%CI (lower-upper)	*P-*value
Age	0.89	0.63–1.27	0.521			
Gender	0.98	0.69–1.4	0.919			
Histological type	2.03	0.66–6.29	0.218			
Histologic grade	0.98	0.8–1.21	0.873			
Stage	1.66	1.38–1.99	0.000	1.11	0.86–1.44	0.416
T classification	1.78	1.49–2.12	0.000	1.65	1.26-2.15	0.000
N classification	0.98	0.68–1.42	0.926			
M classification	1.19	0.8–1.78	0.394			
Radiation therapy	0.75	0.26–2.17	0.592			
Residual tumor	1.27	1.01–1.61	0.042	1.37	1.08–1.74	0.010
SNHG4	2.06	1.37–3.1	0.001	1.95	1.29–2.96	0.002

### SNHG4-related signaling pathway and ceRNA network

**Figure 4:**
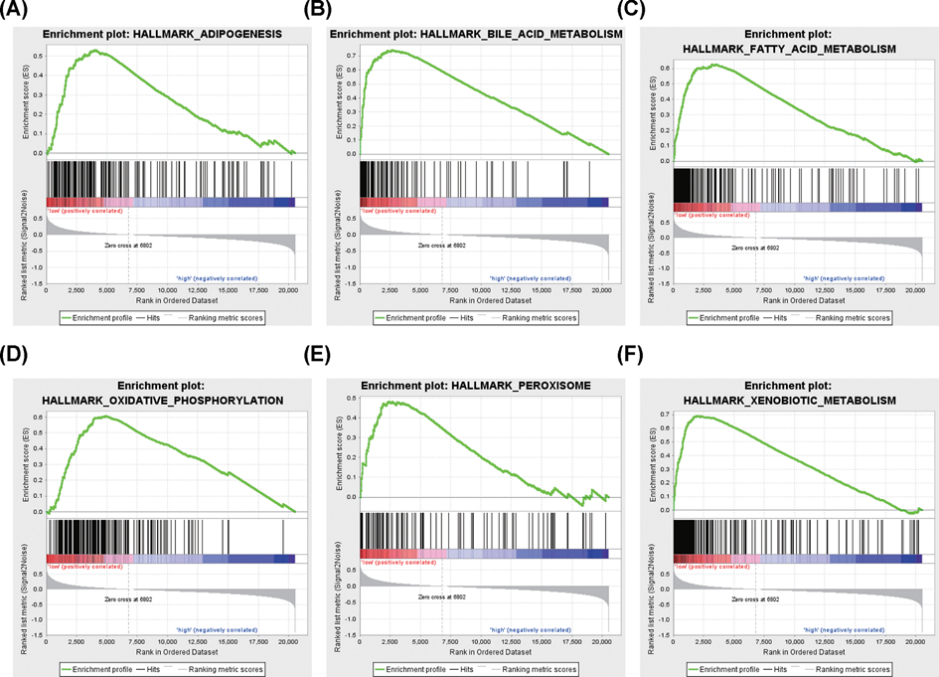
Enrichment plots from GSEA Adipogenesis (**A**), bile acid metabolism (**B**), fatty acid metabolism (**C**), oxidative phosphorylation (**D**), peroxisome (**E**), and xenobiotic metabolism (**F**) are differentially enriched in SNHG4-related liver cancer.

To identify SNHG4-related signaling pathway activated in liver cancer, we conducted the GSEA between low and high SNHG4 expression data sets. Significant differences (FDR < 0.25, NOM *P*-value < 0.05) in the enrichment of MSigDB Collection (h.all.v6.2.symbols.gmt) and the details are shown in Figure 4 and Table 5. Oxidative phosphorylation, adipogenesis, fatty acid metabolism, bile acid metabolism, xenobiotic metabolism, and peroxisome are differentially enriched in SNHG4 expression-related phenotype.

**Table 5:** Gene sets enriched in expression-related phenotype

Gene set	NES	NOM *P*-val	FDR *q*-val
HALLMARK_OXIDATIVE_PHOSPHORYLATION	2.21	0.01	0.01
HALLMARK_ADIPOGENESIS	2.19	0.00	0.00
HALLMARK_FATTY_ACID_METABOLISM	2.14	0.00	0.00
HALLMARK_BILE_ACID_METABOLISM	2.02	0.00	0.01
HALLMARK_XENOBIOTIC_METABOLISM	1.97	0.00	0.01
HALLMARK_PEROXISOME	1.93	0.00	0.01

NES: normalized enrichment score; NOM: nominal; FDR: false discovery rate. Gene sets with NOM *P*-val <0.05 and FDR *q*-val <0.25 are considered as significant.

**Figure 5:**
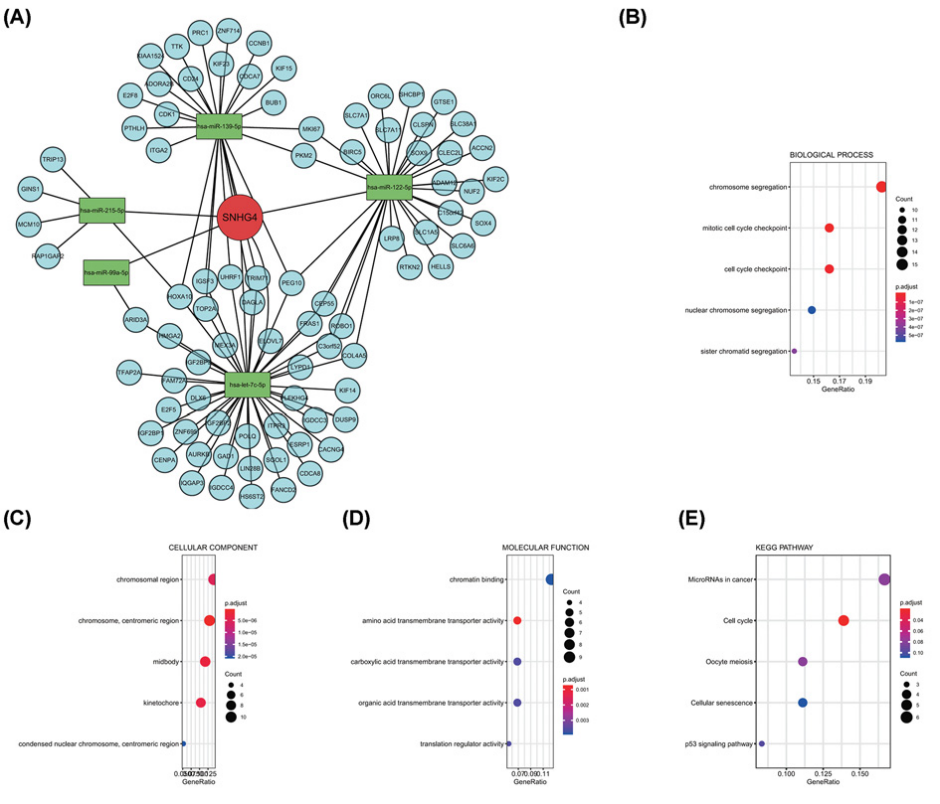
SNHG4-related ceRNA network and related signaling pathway (**A**) SNHG4-related ceRNA network was constructed, the red circle represents the lncRNA SNHG4, green rectangles represent the microRNAs, and the blue circle represents the mRNA. GO biological process (**B**), cellular component (**C**), molecular function (**D**), and KEGG (**E**) enrichment of mRNA in the ceRNA network.

In order to explore the further mechanism of SNHG4, we select 12 down-regulated differentially expression microRNAs (DEMs) and 1142 up-regulated expression encoding genes (DEGs) between low and high SNHG4 expression groups differentially. Next, we merged DEMs, DEGs and predicted miRNA–mRNA targets and conducted the SNHG4-related ceRNA network (Figure 5A). GO and KEGG enrichment analysis revealed the related functions and signaling pathways of mRNAs in the ceRNA network (Figure 5B–E). The correlation between the expression of SNHG4 and mRNAs in the ceRNA network was provided in the Supplementary Information.

## Discussion

Liver cancer is associated with a high mortality rate worldwide. Despite the advance of therapies, the poor prognosis of liver cancer is still inextricability. Therefore, it is important to find reliable biomarkers for diagnosis and prognosis in liver cancer. In recent years, bioinformatics has attracted much attention because of its significance in screening markers. We also have been working on the exploration biomarkers for different types of cancer by bioinformatics [17–31].

In the present study, we found the higher SNHG4 expression in liver cancer, and the relationship between SNHG4 expression and histological type, histologic grade, stage, T classification, and survival status. In the further, we analyzed the effect of SNHG4 on the overall survival and relapse-free survival, and made subgroup analysis to explore it in depth. Besides, we studied the related signaling pathway through GSEA and conducted the SNHG4-related ceRNA network.

Small nuclear RNA host genes (SNHGs), encoded by some lncRNAs, are a class of small RNA molecules and play a role in chemical modifications of other RNAs including rRNAs, tRNAs, and snRNAs. Recently, many studies have found that the aberrant expression of snoRNAs might act as an oncogene in the progression of the tumor. For example, Chen found that SNHG8 overexpressed in non-small cell lung carcinoma (NSCLC) [32], and Dong found that SNHG8 is an oncogene in human hepatocellular carcinoma [33], Meng found SNHG6 promotes glioma tumorigenesis in glioma [34], and Zhu found SNHG4 overexpressed in hepatocellular carcinoma [35]. Consistent with these studies, we found higher SNHG4 expression in liver cancer and it may be a biomarker.

As for the prognostic value of SNHGs, limited studies have deeply explored it. SNHG1, SNHG3, SNHG20 have been separately proved to be a prognostic biomarker in neuroblastoma [36], ovarian cancer [37], and colorectal cancer [38]. Besides, only Zhu made a bioinformatic analysis of lncRNAs and found SNHG4 might be valuable prognostic markers in HCC [35]. In the present study, we also made the same conclusion that SNHG4 expression is an independent predictor of poor prognosis in liver cancer. But we further studied the prognostic value of SNHG4 in the subgroup and found its limitation in female and younger patients, which may contribute to the precision medicine.

Considering for a potential mechanism of SNHG4, related studies were rare. M. Ahmad Chaudhry first found SNHG4 might be involved in the bystander effect, a phenomenon that the irradiated cells communicate with unirradiated cells and induce changes in them [5]. Recently, Rudia Xu found that LncRNA SNHG4 promotes tumor growth by sponging miR-224-3p in osteosarcoma [6]. In the present study, we made the GSEA analysis and found that oxidative phosphorylation, adipogenesis, fatty acid metabolism, bile acid metabolism, xenobiotic metabolism, and peroxisome are differentially enriched in SNHG4 expression-related phenotype. Besides, we conducted the SNHG4-related ceRNA network and provided the clue for the future studies. There are still need to explore the underground mechanism by experiments in this field.

To our knowledge, this is the first study identifying a correlation between the expression level of SNHG4 and the prognosis of patients with liver cancer and exploring related mechanism. The results of our study have shown that the up-regulation of SNHG4 expression is associated with poor survival and has an independent prognostic role in liver cancer. Our study provides a new insight that SNHG4 played a valuable role in the prognosis of liver cancer, which may have an influence on the signaling pathway and laid a foundation for further studies to explore the SNHG4-related ceRNA mechanisms in deep.

## Supplementary Material

Supplementary Figures S1-S2 and Table

## Abbreviations

NSCLC: non-small cell lung carcinoma
OS: overall survival
RFS: relapse-free survival
SNHG4: small nucleolar RNA host gene 4
TCGA: The Cancer Genome Atlas
