# Supplementary material for: The prognostic value of lncRNA SNHG4 and its potential mechanism in liver cancer
Source: Biosci Rep. 2020 Jan 31;40(1):BSR20190729. doi: 10.1042/BSR20190729 (PMC6997108; doi:10.1042/BSR20190729)
Supplement: Supplementary Figures S1-S2 and Table [file BSR-2019-0729_supp.zip › BSR-2019-0729_supp.pdf]

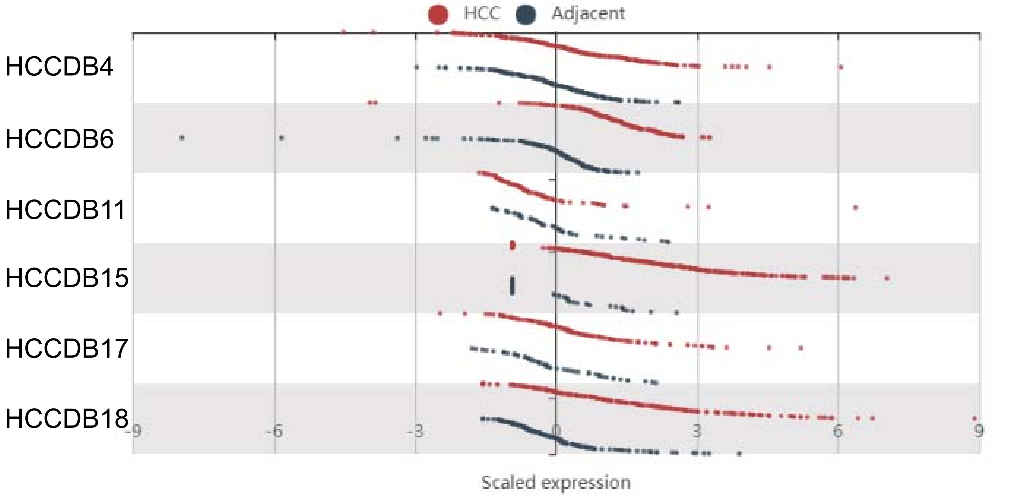
HCC
Adjacent
HCCDB4
HCCDB6
HCCDB11
HCCDB15
HCCDB17
HCCDB18
-9
-6
-3
0
3
6
9
Scaled expression

Figure S1. Differentially SNHG4 expression in HCCDB database.

Differentially SNHG4 expression between HCC and adjacent tissue from HCCDB database.

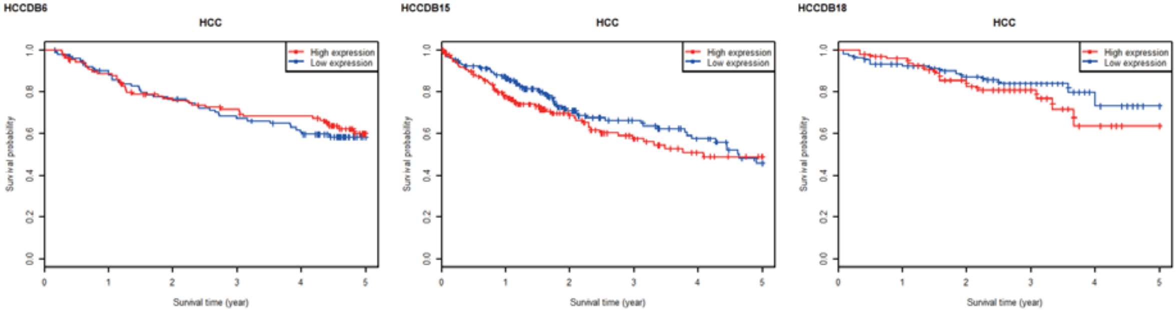
HCCDB6
HCC
High expression
Low expression
Survival probability
0.0
0.2
0.4
0.6
0.8
1.0
0
1
2
3
4
5
Survival time (year)
HCCDB15
HCC
High expression
Low expression
Survival probability
0.0
0.2
0.4
0.6
0.8
1.0
0
1
2
3
4
5
Survival time (year)
HCCDB18
HCC
High expression
Low expression
Survival probability
0.0
0.2
0.4
0.6
0.8
1.0
0
1
2
3
4
5
Survival time (year)

Figure S2. Survival curve grouped by SNHG4 expression in HCCDB database.

Survival curve between SNHG4 high and SNHG4 low group in HCCDB database.
